# NRF2 Protects against Altered Pulmonary T Cell Differentiation in Neonates Following In Utero Ultrafine Particulate Matter Exposure

**DOI:** 10.3390/antiox11020202

**Published:** 2022-01-21

**Authors:** Carmen H. Lau, Drew Pendleton, Nicholas L. Drury, Jiayun Zhao, Yixin Li, Renyi Zhang, Gus A. Wright, Aline Rodrigues Hoffmann, Natalie M. Johnson

**Affiliations:** 1 Department of Veterinary Pathobiology, Texas A&M University, College Station, TX 77843, USA; clau@cvm.tamu.edu (C.H.L.); gwright@cvm.tamu.edu (G.A.W.); 2 Department of Environmental and Occupational Health, Texas A&M University, College Station, TX 77843, USA; drew976@tamu.edu (D.P.); ndrury@cvm.tamu.edu (N.L.D.); 3 Department of Chemistry, Texas A&M University, College Station, TX 77843, USA; jyzhao1218@tamu.edu (J.Z.); yxli11@tamu.edu (Y.L.); renyi-zhang@tamu.edu (R.Z.); 4 Department of Atmospheric Sciences, Texas A&M University, College Station, TX 77843, USA; 5 Flow Cytometry Facility, Texas A&M University, College Station, TX 77843, USA; 6 Department of Comparative, Diagnostic, and Population Medicine, University of Florida, Gainesville, FL 32653, USA; aline.hoffmann@ufl.edu

**Keywords:** air pollution, ultrafine particulate matter, prenatal exposure, *Nrf2*, oxidative stress, knockout model, pulmonary immunophenotype

## Abstract

Early life exposure to particulate matter (PM) air pollution negatively impacts neonatal health. The underlying mechanisms following prenatal exposure, particularly to ultrafine particles (UFP, diameter ≤ 0.1 μm), are not fully understood; To evaluate the role of *Nrf2* in response to in utero UFP exposure, we exposed time-mated *Nrf2*-deficient (*Nrf2*^−/^^−^) or wildtype (WT) mice to filtered air (FA) or 100 μg/m^3^ ultrafine PM daily throughout pregnancy. Offspring were evaluated for pulmonary immunophenotypes and pulmonary/systemic oxidative stress on postnatal day 5, a timepoint at which we previously demonstrated viral respiratory infection susceptibility; *Nrf2*^−/^^−^ offspring exposed to FA had significantly lower average body weights compared to FA-exposed WT pups. Moreover, PM-exposed *Nrf2*^−/^^−^ offspring weighed significantly less than PM-exposed WT pups. Notably, PM-exposed *Nrf2*^−/^^−^ offspring showed a decreased pulmonary Th1/Th2 ratio, indicating a Th2 bias. Th17 cells were increased in FA-exposed *Nrf2*^−/^^−^ neonates yet decreased in PM-exposed *Nrf2*^−/^^−^ neonates. Analysis of oxidative stress-related genes in lung and oxidative stress biomarkers in liver tissues did not vary significantly across exposure groups or genotypes. Collectively, these findings indicate that the lack of *Nrf2* causes growth inhibitory effects in general and in response to gestational UFP exposure. Prenatal UFP exposure skews CD4+ T lymphocyte differentiation toward Th2 in neonates lacking *Nrf2*, signifying its importance in maternal exposure and infant immune responses.

## 1. Introduction

Early life exposure to particulate matter (PM) air pollution is linked with numerous adverse developmental outcomes, impacting global neonatal morbidity and mortality, and increasing risk for chronic health effects later in life [1,2]. PM is classified by size into coarse (PM_10_, <10 µm), fine (PM_2.5_, <2.5 µm), and ultrafine particles (UFPs, diameter < 0.1 µm). The World Health Organization recently lowered its guideline values for PM_10_ and PM_2.5_ aimed at promoting reduced risks for acute and chronic health effects [3]. Conversely, no guidelines or regulations exist for UFPs. The small diameter of the UFPs allows for a deeper penetration into airways, the ability to translocate systemically, and a higher surface area to volume ratio that allows increased absorption of potentially toxic chemicals generating systemic oxidative stress responses [4]. Evidence from experimental models and human placentae demonstrates UFPs can cross the placental barrier into fetal circulation [5,6].

Since fetal development is a period of rapid differentiation and growth, in utero exposure to UFPs, and also more broadly PM, represents a unique window of susceptibility. Prenatal exposure to PM_10_ and PM_2.5_ has been consistently linked with preterm birth, infant low birth weight [7,8,9], and respiratory morbidities, including risks for respiratory infection and childhood asthma [10,11,12]. Emerging evidence supports prenatal UFP exposure is independently linked with childhood asthma incidence [13,14]. Additional observation studies demonstrate early life exposure to various PM fractions, including organic carbon and sulfates, and particles in size fractions of 5–560 nm mainly from traffic emissions, are associated with increased risk of hospitalization for respiratory infections among children in the U.S. and in China [15,16]. In the latter study, significant associations of respiratory emergency room visits were also found to be associated with secondary aerosols and emissions from gasoline and diesel vehicles. Findings from a birth cohort study in Korea demonstrate increased susceptibility of lower respiratory tract infections in infants prenatally exposed to PM_2.5_ and second-hand smoke was significantly modified by polymorphisms in maternal genes related to oxidative stress response pathways, especially in the nuclear factor erythroid 2-related factor (*Nrf2*) gene.

*Nrf2* regulates and enhances the cellular response to oxidative stress [17]. A transcription factor in the Cap n’ Collar family with a basic leucine zipper, *Nrf2* is bound in the cytoplasm by the Keap1-Cul3 complex [18,19]. In traditional canonical activation, oxidative stress initiates the oxidization of cysteine residues of the dimerized Keap1 protein, altering its conformation to prevent it from binding *Nrf2*. The freed *Nrf2* translocates to the nucleus, where it binds an antioxidant response element (ARE) upstream numerous antioxidant-related genes [18]. In experimental models, disruption of *Nrf2* has been shown to enhance susceptibility to allergic airway inflammatory responses induced by chronic exposure to diesel exhaust particulate matter [20]. While the role of *Nrf2* in standard, adult exposure models is well-studied, the impact of *Nrf2* signaling in maternal and fetal responses to in utero PM exposure, especially the ultrafine fraction, is yet to be clarified.

Therefore, to evaluate the effect of UFP exposure during pregnancy on neonatal outcomes in a model lacking an appropriate oxidative stress response, we exposed *Nrf2* deficient (*Nrf2*^−/^^−^) and wildtype (WT) dams to filtered air (control) or aerosolized ultrafine PM containing diesel exhaust. We evaluated the neonatal pulmonary immunophenotype and state of oxidative stress.

## 2. Methods and Materials

### 2.1. Ultrafine Particle Exposure

Diesel Particulate Matter Standard Reference Material (SRM 2975) was purchased from the National Institute of Standards and Technology (Gaithersburg, MD, USA). All other chemicals and reagents used were obtained commercially at the highest available purity. PM generation followed methods developed by Rychlik et al. [21], with adaptations to accommodate individual housing within whole body exposure chambers, as described by Behlen et al. [22] Briefly, HEPA filtered air was continuously pumped into two separate chambers (FA and PM) where pregnant dams were individually housed. For the PM chamber, UFPs were generated using a custom-built constant output atomizer. We employed a multicomponent aerosol mixture consisting of ammonium nitrate, ammonium sulfate, diesel exhaust PM (NIST, SRM 2975), and potassium chloride, with the mass fractions of 44, 39, 10, and 7%, respectively. Real-time particle size distribution was monitored using a differential mobility analyzer (DMA) in tandem with a condensation particle counter (CPC). The flowrate of HEPA-filtered air was adjusted to ensure consistent particle concentrations ~100 µg/m^3^ within chambers throughout the exposure duration. This dose was selected based on previous findings from our research showing neonatal immune suppression [21] and marked placental changes [22].

*Nrf2*-deficient mice (*Nrf2*^−/^^−^) on C57Bl/6J background were obtained from Dr. Tom Kensler, which were generated as previously reported [23]. Genotyping for homozygous wildtype (*Nrf2*^+/+^) and null (*Nrf2*^−/^^−^) mice was carried out following established methods (see supplemental methods). Mice were housed in a climate-controlled room with 12/12 h light/dark cycle at an AAALAC approved facility at Texas A&M University. All procedures were approved by the Institutional Animal Care and Use Committee of Texas A&M University #2019-0025. Mice had access to standard chow, 19% protein extruded rodent diet (Teklad Global Diets), and water ad libitum except during exposure periods. A schematic of our exposure timeline is shown in Supplemental Figure S1. Male and female WT or *Nrf2*^−/^^−^ mice were time-mated. The presence of a vaginal plug defined gestational day (GD) 0.5. Beginning on GD0.5, dams were randomized and placed into exposure chambers where they were exposed to either FA (n = 5;5) or PM (n = 4;7), listed as (n = WT; *Nrf2*^−/^^−^), respectively, from 0800 to 1400 h (6 h) daily through GD18.5. Following exposure on GD18.5, mice were removed to individual housing and allowed to deliver spontaneously, denoted as PND1 (post-natal day). Pups were weighed and euthanized on PND5 for baseline evaluations.

### 2.2. Flow Cytometry

PND5 lungs were perfused retrograde with sterile phosphate buffered saline for removal of red blood cells, mixed with a Miltenyi Biotic Flow Cytometry kit (Auburn, CA, USA), dissociated with gentleMACS Octo Dissociator (Miltenyi Biotec, Auburn, CA, USA), and strained through a 40-µm cell strainer (MACS SmartStrainers, Miltenyi Biotec, Auburn, CA, USA). The single cell lung suspension was treated with RBC lysis buffer, washed, placed in 90% FBS and 10% DMSO freezing media, and frozen in a Mister Frosty (ThermoFisher (Waltham, MA, USA), cat#: 5100-0001) down to −80 °C and held there until later processing. Samples were combined within litters and sexes to achieve appropriate cellularity. Processing was performed in a 96 well plate. Cells were incubated with Golgi Plug for 5 h, washed with cell staining buffer, blocked with anti-CD16/CD32 for 10 min, and incubated with cell surface fluorescent monoclonal antibodies for 20 min with the panels listed below. Cells were washed twice with cell staining buffer, resuspended in permeabilization wash buffer, incubated with intracellular fluorescent antibodies for 30 min, then washed in buffer twice again. Cells were resuspended in cell staining buffer and analyzed. All antibodies were obtained from BioLegend. T cell staining used Alexa Fluor 488 anti-mouse CD3 [100210] and PE anti-mouse CD69 [104507]. Th1 cell staining used Alexa Fluor 647 anti-rat IFN-γ [507809], APC/Fire 750 anti-mouse CD4 [100460], and PE/Cy7 anti-mouse CD8a [100722]. Th2 cell staining used Brilliant Violet 421 anti-mouse IL-4 [504119]. T regulatory cell staining used Alexa Fluor 647 anti-mouse CD25 [102019] and Brilliant Violet 421 anti-mouse FOXP3 [126419]. FMOs and positive and negative compensation beads were used to set gates for the above populations. Immunostained cells were run on a Luminex/Amnis Cell Stream flow cytometer outfitted with 405 nm, 488 nm, and 640 nm lasers and a 96-well plate autosampler. The BV421 was excited using a 405 nm laser and emission detected using a 448/59 nm bandpass filter. The Alexa Fluor 488, and PE- Cy7 were excited using the 488nm laser and emissions were detected using 513/26 nm, and 795/70 nm bandpass filters, respectively. The Alexa Fluor 647 and APC-Fire 750 were excited using a 640 nm laser and emission detected with a 671/30 nm and 795/70 nm bandpass filters, respectively. The Live/Dead fixable Red stain was excited using the 488 nm laser and emission detected using a 620/29 nm bandpass filter. The samples were run at a flow rate less than 3000 events per second. The flow cytometry data was analyzed using Cell Stream analysis software (Luminex/Amnis). Gating strategies are depicted in supplemental figures (Supplemental Figures S4 and S5).

### 2.3. Gene Expression

Total RNA was extracted from PND5 lungs using TRIzol reagent according to the manufacturer’s protocol (ThermoFisher Scientific). RNA was quantified with a DeNovix DS-11 FX+ Spectrophotometer/Fluorometer with ≥ 1.8 260/280 nm absorbance values. Following purification and standardization of RNA concentration at 100 ng/µL, cDNA was reverse transcribed (Qiagen QuantiTect^®^ Reverse Transcription Kit, Germantown, MD, USA), and transcription levels of key genes related to oxidative stress were analyzed using SYBR Green qRT-PCR (Applied Biosystems™ Power SYBR™ Green PCR Master Mix, Waltham, MA, USA) on a Roche LightCycler^®^ 96 System. Relative expression was calculated using the 2^−ΔΔCt^ method with *Gapdh* as the reference gene.

### 2.4. Thiol Redox Analysis

#### 2.4.1. Sample Preparation

Thiol redox analysis followed Jones and Liang with slight modifications [24]. PND5 livers were snap frozen in liquid nitrogen until analysis. Approximately 10–50 mg of liver tissue was quickly weighed before being homogenized in 1 mL of buffer solution containing an internal standard, γ-glutamylglutamate (γ-Glu-Glu) and centrifuged at 10,000× *g* for 10 min at 4 °C. Then 60 μL of 7.4 mg/mL iodoacetic acid (IAA) was added to 300 μL of the supernatant and immediately vortexed. pH was adjusted to ~9.0 ± 0.2 with ~425 μL of KOH/tetraborate to precipitate proteins, then incubated at room temperature for 30 min before pH was confirmed. 300 µL dansyl chloride (DC) solution (20 mg/mL in acetone) was added to each sample, vortexed, and incubated at room temperature in complete darkness for 16–28 h. 500 µL of HPLC-grade chloroform was added to samples, followed by vortexing and centrifugation. The upper aqueous layer was stored in −80 °C until HPLC analysis.

#### 2.4.2. HPLC Analysis

Samples were thawed on ice, centrifuged for 10 min, and aliquots were transferred to an HPLC autosampler vial. 35 µL was injected on a Supelcosil LC-NH2 column with internal dimensions of 5 µm, 4.6 mm × 25 cm (Supelco, Bellefonte, PA, USA). Column oven operating temperature was held constant at 35 °C. HPLC mobile phases included solvent A (80% *v*/*v* methanol/water) and solvent B (acetate-buffered methanol). Initial solvent conditions were 80% A, 20% B at 1 mL/minute for 10 min. A linear gradient to 20% A and 80% B was then run from 10–30 min. From 30 to 35 min, the flow gradient was maintained at 20% A and 80% B. From 35 to 42 min, conditions were returned to 80% A and 20% B. Detection of desired thiols was obtained by fluorescence monitoring. Approximate elution time frames of compounds of interest were as followed: cystine (CySS) from 9 to 9.5 min; cysteine (Cys) from 10 to 10.5 min; γ-GluGlu from 12 to 13 min; glutathione (GSH) from 19 to 19.5 min; and glutathione disulfide (GSSG) from 23.5 to 24 min.

### 2.5. Statistical Analysis

Statistical analysis was performed using Prism (v8, GraphPad Software, San Diego, CA, USA) to determine differences in offspring outcomes based on exposure group. Two-way analysis of variance (ANOVA) with Tukey’s multiple comparisons tests were conducted. An adjusted *p* value of < 0.05 was considered statistically significant.

## 3. Results

### 3.1. In Utero UFP Exposure Alters Neonatal Weights in *Nrf2*^−/^^−^ Mice

**Figure 1:**
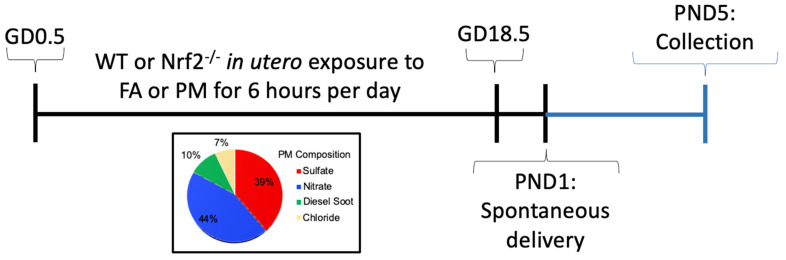
Experimental timeline for mouse exposure model. GD: gestation day; FA: filtered air; PM: particulate matter; PND: postnatal day; WT: wildtype.

**Figure 2:**
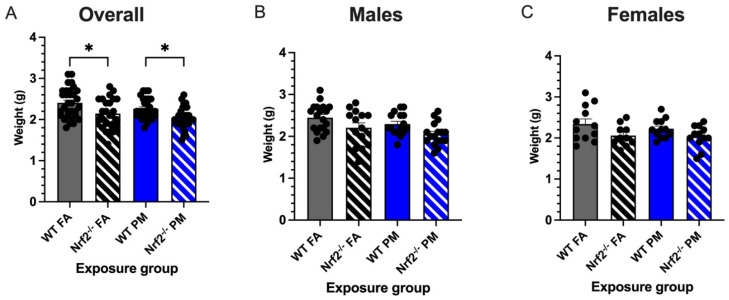
Neonatal weights measured at postnatal day (PND) 5 (21 litters with total n = 112). (**A**) Overall, *Nrf2*^−/^^−^ offspring showed significantly decreased body weights from their wildtype counterparts in both PM- and FA-exposed groups. Male (**B**) and female (**C**) offspring weight changes did not vary significantly. Offspring sample sizes, listed as (n = Male, Female), from 4–7 litters, include WT FA (n = 19,12), WT PM (n = 16,12), *Nrf2*^−/^^−^ FA (n = 14,12), and *Nrf2*^−/^^−^ PM (n = 17,12). Error bars represent SEM. Data analyzed using two-way ANOVA with Tukey’s multiple comparison test. * *p* < 0.05.

Low birth weight is one of the common perinatal effects in children born to mothers exposed to particulate matter air pollution [2,7]. We monitored birth weights in neonates born to *Nrf2*^−/^^−^ and wildtype (WT) dams exposed for the length of gestation (Figure 1) to either filtered air (FA, 0 μg/m^3^) or ultrafine particulate matter (PM, 111.87 ± 4.40 μg/m^3^, mean ± SEM) (Supplemental Figure S1). Regardless of exposure group or litter size, no statistical significance was detected between the average daily maternal weight gain during gestation (*p* = 0.560) or the final weights of the pregnant dams before parturition (*p* = 0.570) (Supplemental Figure S2). Average litter sizes did not vary between exposures or genotypes (*p* = 0.809), nor was there any correlation between litter size and pup weight (*p* = 0.600) (data not shown). Initial weights of pups taken on PND5 showed *Nrf2*^−/^^−^ pups exposed to FA with significantly reduced weight gain compared to WT FA-exposed pups (*p* = 0.0001) (Figure 2). Moreover, PM-exposed *Nrf2*^−/^^−^ offspring weighed significantly less than PM-exposed WT pups (*p* = 0.045). These changes failed to reach statistical significance when separated by sex.

### 3.2. *Nrf2*^−/^^−^ Neonates Exhibit Th2 Pulmonary Immune Bias

Prenatal PM exposure is linked to a higher incidence of childhood asthma and respiratory infections, often driven by or compounded by a Th2-skewed immune response [10,25]. To evaluate the differences in neonatal pulmonary immunity between *Nrf2*^−/^^−^ and WT FA- or PM-exposed mice, lungs were taken at PND5, and both a T regulatory panel and a Th1-Th2 panel was evaluated all in samples to examine the effect that UFP exposure would elicit from the immune system, particularly within its lymphocytes. The Th1 and Th2 panel assessed multiple markers: CD3, CD4, CD8, IFN-γ, IL-4, and IL-17A. From these, a Th1/2 ratio was determined to look for T helper cell bias. Overall CD4+ cell levels modestly, though not significantly, decreased in the *Nrf2*^−/^^−^ PM group (*p* = 0.100); CD8+ cells did not show any significant trends (*p* = 0.315) (Supplemental Figure S3).

**Figure 3:**
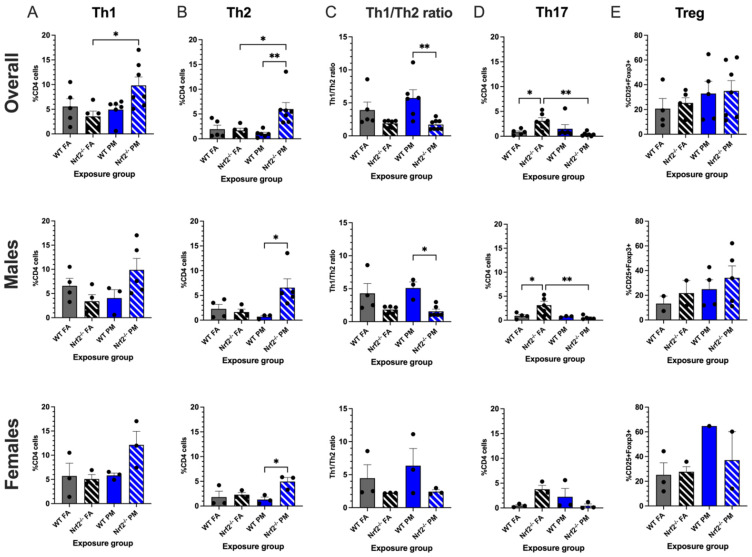
Flow cytometry analysis of neonatal lungs at postnatal day (PND) 5. Percentages of CD4+ cells with Th1 (**A**) and Th2 (**B**) differentiation were significantly increased in *Nrf2*^−/^^−^ PM-exposed mice. (**C**) *Nrf2*^−/^^−^ PM-exposed mice demonstrated a significantly lower Th1/2 ratio, indicating a Th2 bias. (**D**) Th17 cells were significantly increased in FA-exposed *Nrf2*^−/^^−^ mice. (**E**) T regulatory cells did not differ significantly across groups. Th1/2 offspring sample sizes, listed as (n = Male, Female), from 4–7 litters, include WT FA (n = 3,3), WT PM (n = 3,3), *Nrf2*^−/^^−^ FA (n = 5,3), and *Nrf2*^−/^^−^ PM (n = 6,3). Treg offspring sample sizes, listed as (n = Male, Female), from 4-7 litters, include WT FA (n = 2,3), WT PM (n = 4,1), *Nrf2*^−/^^−^ FA (n = 2,3), and *Nrf2*^−/^^−^ PM (n = 5,2). Some samples contained both male and female to maximize cell usage, and are represented in the overall graphs; therefore, combined male and female numbers may not equal overall numbers. Error bars represent SEM. Data analyzed using two-way ANOVA with Tukey’s multiple comparison test. (* *p* < 0.05; ** *p* < 0.01).

*Nrf2*^−/^^−^ PM-exposed neonates showed the highest numbers of both Th1 CD4+ cells, (positive for IFN-γ) and Th2 CD4+ cells (positive for IL-4). Within the Th1 subset, the combined data demonstrated that the *Nrf2*^−/^^−^ PM CD4+ average of 9.83 ± 1.71% was significantly greater than the 3.55 ± 1.07% average of the *Nrf2*^−/^^−^ FA group (*p* = 0.025), a trend is also reflected in the sex-separated data (Figure 3A). The *Nrf2*^−/^^−^ PM males and females averaged 9.87 ± 2.38% and 12.15 ± 2.76%, respectively, as compared to the lower *Nrf2*^−/^^−^ FA males (3.40 ± 1.36%) and females (5.09 ± 0.92%). Within the Th2 subset, the *Nrf2*^−/^^−^ PM-exposed neonates, when combined, had significantly higher numbers of Th2 cells than the *Nrf2*^−/^^−^ FA-exposed (*p* = 0.016), WT PM-exposed (*p* = 0.003), and WT FA-exposed groups (*p* = 0.025) (Figure 3B). The number of *Nrf2*^−/^^−^ PM Th2 cells was significantly higher than the WT PM group in both males (6.57 ± 1.79% vs. 1.64 ± 0.58%, *p* = 0.037) and females (4.95 ± 0.83% vs. 2.30 ± 0.45%, *p* = 0.050).

These averages led the Th1/2 ratio to have a distinct separation between WT and *Nrf2*^−/^^−^ mice, wherein the ratio of the WT groups was greater than those of the *Nrf2*^−/^^−^ groups, which was significant in the PM exposed group (Figure 3C). A higher Th1/2 ratio represents skewing of the immune system towards a Th1 response, with the lower ratio conversely representing a Th2-biased immune response. Thus, the *Nrf2*^−/^^−^ neonates display a Th2 immune bias by the *Nrf2*^−/^^−^ PM ratio of 1.71 ± 0.27%, decreased in comparison to the WT PM ratio of 5.73 ± 1.26% (*p* = 0.002) but similar to the *Nrf2*^−/^^−^ FA ratio of 1.90 ± 0.22%. The males’ ratios follow similar patterns at 1.56 ± 0.34%, 5.10 ± 0.91%, and 1.83 ± 0.26%, respectively (*p* = 0.002), but the female ratios showed no significant differences (*p* = 0.395).

Th17 CD4+ cells are most commonly recognized for their role in autoimmune phenotypes. As seen in Figure 3D, this subset demonstrated the most varied trends of the CD4+ cells, with an unexpectedly high count in the *Nrf2*^−/^^−^ FA males (3.14 ± 0.80%) and females (3.77 ± 0.76%), with a combined average of 3.10 ± 0.66%. This combined average was significantly higher than the *Nrf2*^−/^^−^ PM and WT FA groups (*p* = 0.008 and *p* = 0.047, respectively), and the males in this group significantly exceeded their *Nrf2*^−/^^−^ PM (*p* = 0.009), WT FA (*p* = 0.036), and WT PM (*p* = 0.040) counterparts. These findings may suggest a constitutively high level of Th17 cells in the *Nrf2*^−/^^−^ genotype that is suppressed by PM exposure. The T regulatory panel overall did not show any significant differences between groups in both genotypes (Figure 3E).

### 3.3. Expression of Oxidative Stress-Related Genes in Pulmonary Tissue

**Figure 4:**
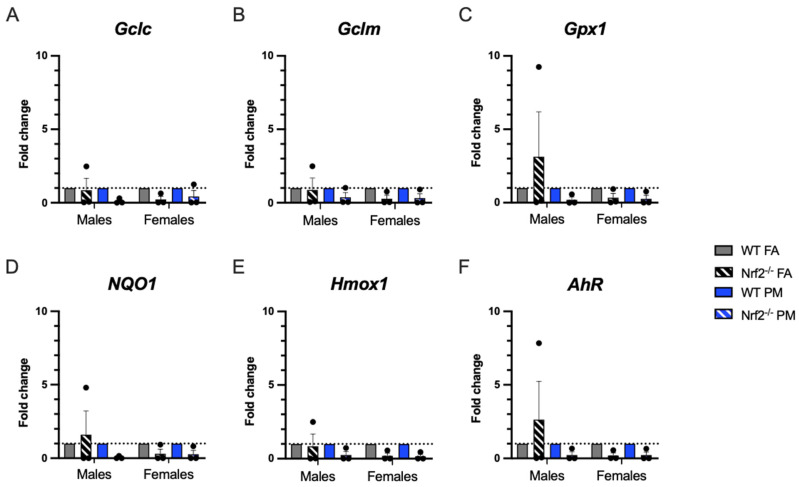
Pulmonary gene expression of oxidative stress-related genes. Offspring sample sizes, listed as (n = Male, Female), from 4–7 litters, include WT FA (n = 3,3), WT PM (n = 3,3), *Nrf2^−/^^−^* FA (n = 3,3), and *Nrf2^−/^^−^* PM (n = 3,3). Data was standardized to the mean of the WT FA group or WT PM group, respetively, therefore, no data points are shown for those groups. Error bars represent SEM. Data analyzed using two-way ANOVA with Tukey’s multiple comparison test.

Quantitative real-time PCR (qPCR) was performed in PND5 lungs to evaluate oxidative stress-related gene expression (Figure 4). We expected that *Nrf2*^−/^^−^ neonates would have less ability to respond to oxidative stress and that PM exposure would enhance this impaired response. Overall, there were no significant differences in expression of selected genes. A few of the interesting trends in the male *Nrf2*^−/^^−^ may be driven by sample variability.

### 3.4. Thiol Ratios in Hepatic Tissue

**Figure 5:**
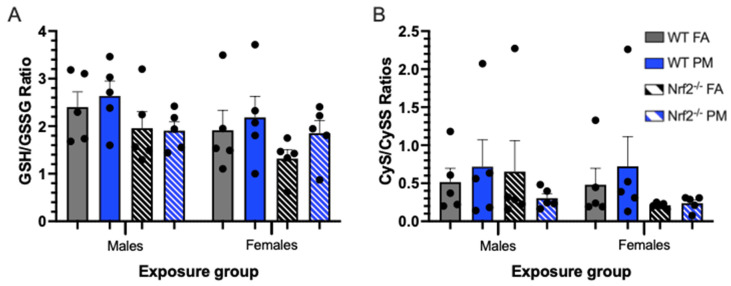
Oxidative stress biomarkers depicting thiol ratios of glutathione (GSH) to glutathione disulfide (GSSG) (**A**) and cysteine (CyS) to cystine (CySS) (**B**) in neonatal livers at postnatal day (PND) 5, representing systemic oxidative stress. Higher ratios indicate an increased capacity for reduction of reactive oxygen species. Though not significantly different, *Nrf2*^−/^^−^ neonates generally have lower thiol ratios, and thus lower redox capacity, than their WT counterparts, and PM-exposed neonates generally have increased thiol ratios versus their FA-exposed counterparts. Offspring sample sizes, listed as (n = Male, Female), from 3 litters, include WT FA (n = 5,5), WT PM (n = 5,5), *Nrf2*^−/^^−^ FA (n = 5,5), and *Nrf2*^−/^^−^ PM (n = 5,5). Error bars represent SEM. Data analyzed using two-way ANOVA with Tukey’s multiple comparison test.

We extrapolated the neonatal systemic oxidative stress state by measuring thiol redox capacity in PND5 livers by HPLC analysis. The ratios of glutathione (GSH) to glutathione disulfide (GSSG), and cysteine (Cys) to cystine (CySS) indicate the neonates’ ability to reduce reactive oxygen species (ROS) by donating an electron from either GSH or Cys. Both indicators showed males and females were not significantly different (Table S1, Figure 5).

## 4. Discussion

PM exposure confers significant detriments to human health, particularly during susceptible life stages, such as fetal development, with consequent negative health effects during infancy and childhood. The report on the State of Global Air 2020, included for the first time the burden of neonatal mortality from PM_2.5_ exposure, largely driven through preterm birth and low infant birth weight [2]. Moreover, it was estimated that air pollution also contributed to as much as 30% of lower-respiratory infections. Our previous research demonstrated offspring pulmonary immune suppression due to in utero UFP exposure with implications for infection risk [21]. The role of the current study was to elucidate the mechanisms by which in utero UFP exposure may predispose the neonatal immunophenotype to a more susceptible state. NRF2 is a key transcription factor that regulates over 1000 other genes and proteins to produce a protective antioxidant and anti-inflammatory effect [18]. By incorporating a *Nrf2* knockout mouse model into our established exposure paradigm, we demonstrate *Nrf2* deficiency impacts birth weight and neonatal immune response.

Birth weight can be used as an indicator of health in the neonate, during childhood, and even into adulthood, as low birth weight often accompanies numerous other comorbidities. Uwak et al., performed a meta-analysis demonstrating associations of prenatal PM exposure and low birth weight in numerous human case-control and cohort studies [7]. Our mouse model demonstrated that the lack of *Nrf2* caused growth inhibitory effects that was observed in response to both FA (filtered air control) and PM exposure. Previous evidence shows that *Nrf2* knockout mice had decreased fetal weights due to decreased placental efficiency [26]. However, Nezu et al., found no difference in fetal weights when *Nrf2* was nullified in their preeclampsia pregnant mouse model [27]. Interestingly, PM exposure on its own (in WT mice) did not result in impaired offspring weight. This is substantiated by evidence from our previous models using WT mice [21]. There is a significant difference in neonatal weight between PM-exposed WT and *Nrf2*^−/^^−^ mice, further signifying the importance of *Nrf2* in both basal conditions, as well as following an environmental challenge.

*Nrf2* is also known to play a role in adaptive immune responses. A baseline pulmonary immunophenotype of the neonates was carried out to determine T cell status following early life exposure to PM. No significant differences in CD8+ or CD4+ cells were observed between any of the groups in combined or sex-separated data. Adult *Nrf2*^−/^^−^ mice also did not demonstrate differences in CD8+ cell expression in a previous study [28]. CD4+ subsets in our model had several notable changes though. In particular, PM-exposed *Nrf2*^−/^^−^ neonates surpassed all other groups in increased levels of Th1 and Th2 differentiated CD4+ cells. They presented a phenotype with a prominent Th2-biased immune system, which can potentially predispose neonates to having more severe respiratory inflammation if challenged with viruses or allergens. Yang et al., demonstrated an association to prenatal indoor PM_2.5_ and tobacco smoke exposure and increased respiratory tract infections in children, potentially modified by maternal *Nrf2* status [29]. Two studies conducted by Perveen et al. indicate a correlation in altered cord blood T cells and the risk of the neonate developing allergic-type symptoms later in life [30,31]. These cord blood cells may be the first step in elucidating the priming of the neonatal pulmonary T cell differentiation.

The balance between autoimmunity and immunosuppression is regulated by Th17 and T regulatory cells, respectively. Abnormal or deficient *Nrf2* signaling has been documented as a contributor in some autoimmune diseases, such as asthma or multiple sclerosis [32]. Possible mechanisms of functional *Nrf2* protecting against autoimmunity range from suppression of Th1 and Th17 cells to the reduction of oxidative stress that may potentially reduce the production of autoimmune self-antigens [32]. Adult *Nrf2* deficient mice frequently develop lupus-like autoimmune diseases [28]. In this study, we did not observe a significant impact on levels of Treg cells in the PM-exposed neonates of both genotypes. Within the Th17 cells, we observed that the lack of *Nrf2* increased the percentage of this subset in neonates; however, this effect was mitigated in the PM-exposed offspring, a finding that was also noted in our previous work by Rychlik et al., when offspring were challenged with house dust mite [21]. This may suggest the propensity of the *Nrf2* knockout mouse towards inflammatory diseases naturally, with a potential mitigation of these effects by the PM. Notably, response to a viral challenge could still be of substantial impact due to these immune alterations.

PM exposure is also documented to generate reactive oxygen species and create a state of oxidative stress observed in both humans and animal models. We evaluated six oxidative stress-related genes to determine if oxidative stress is a prominent mechanism by which UFPs exert their detrimental influence and which could be exacerbated by the lack of *Nrf2* [33]. The three genes related to glutathione (*Gclc, Gclm, Gpx1*) generally demonstrated lower expression in the *Nrf2* deficient neonates, as expected, particularly in the females. *Hmox1* demonstrated similar patterns. Both *NQ01* and *AhR* demonstrated increased levels of expression in PM exposed mice in both genotypes, indicating a response to oxidative stress in our model. However, in the *Nrf2* FA-exposed males, all oxidative stress related genes had a high level of constitutive expression, with the expression in *AhR*, *NQ01*, and *Gpx1* exceeding those levels of even the WT FA-exposed males. The high expression of *AhR* may account for the increased expression of the other related genes, as this gene can induce many other pathways, including upregulation of *Nrf2* in WT mice [34,35]. This finding exhibits a distinct sex specific response of the *Nrf2* male neonates as compared to the females, which will be crucial in interpreting future studies using *Nrf2* deficient neonates.

By utilizing hepatic tissue, we evaluated the systemic state of oxidative stress in the offspring compared to the pulmonary state. Overall, there were not significant differences in oxidative stress biomarkers, as measured by thiol ratios of reduced/oxidized glutathione and its precursor cysteine. These results suggest that at a systems level in utero PM exposure, without functional *Nrf2* signaling, dose not manifest as altered GSH/GSSG or Cys/CySS ratios. Additional markers of oxidative stress, in lung and liver tissues should be investigated further. Moreover, the source of PM may more readily impact these pathways and warrants additional hazard assessment.

## 5. Conclusions

We demonstrated that the lack of *Nrf2* leads to growth inhibitory effects basally and upon prenatal exposure to UFPs. Importantly, prenatal UFP exposure skews CD4+ T lymphocyte differentiation toward Th2 in neonates lacking *Nrf2*, signifying its importance in maternal exposure and infant immune responses.

## Data Availability

All data is presented in the article and supplementary materials.

## Supplementary Materials

The following supporting information can be downloaded at: https://www.mdpi.com/article/10.3390/antiox11020202/s1, Figure S1: Experimental timeline and UFP composition, Figure S2: Maternal average daily UFP exposure. Figure S3: Maternal gestational weights. Figure S4: CD8+ and CD4+ T cell flow cytometry. Figure S5: CD4+ T cell subset gating strategies. Figure S5: T regulatory gating strategy. Table S1: Oxidative stress biomarkers. Genotyping methods.
