# Supplementary material for: NRF2 Protects against Altered Pulmonary T Cell Differentiation in Neonates Following In Utero Ultrafine Particulate Matter Exposure
Source: Antioxidants (Basel). 2022 Jan 21;11(2):202. doi: 10.3390/antiox11020202 (PMC8868442; doi:10.3390/antiox11020202)
Supplement: Supplementary file 1 [file antioxidants-11-00202-s001.zip › antioxidants-1511587-supplementary.pdf]

Supplementary Materials for

NRF2 Protects against Altered Pulmonary T Cell Differentiation in Neonates Following In Utero Ultrafine Particulate Matter Exposure

Carmen H. Lau [1], Drew Pendleton [2], Nicholas L. Drury [2], Jiayun Zhao [3], Yixin Li [3], Renyi Zhang [3,4], Gus A. Wright [1,5], Aline Rodrigues Hoffmann [6], Natalie M. Johnson [3,*]

* Correspondence: nmjohnson@tamu.edu; Tel.: 1+979-436-9325

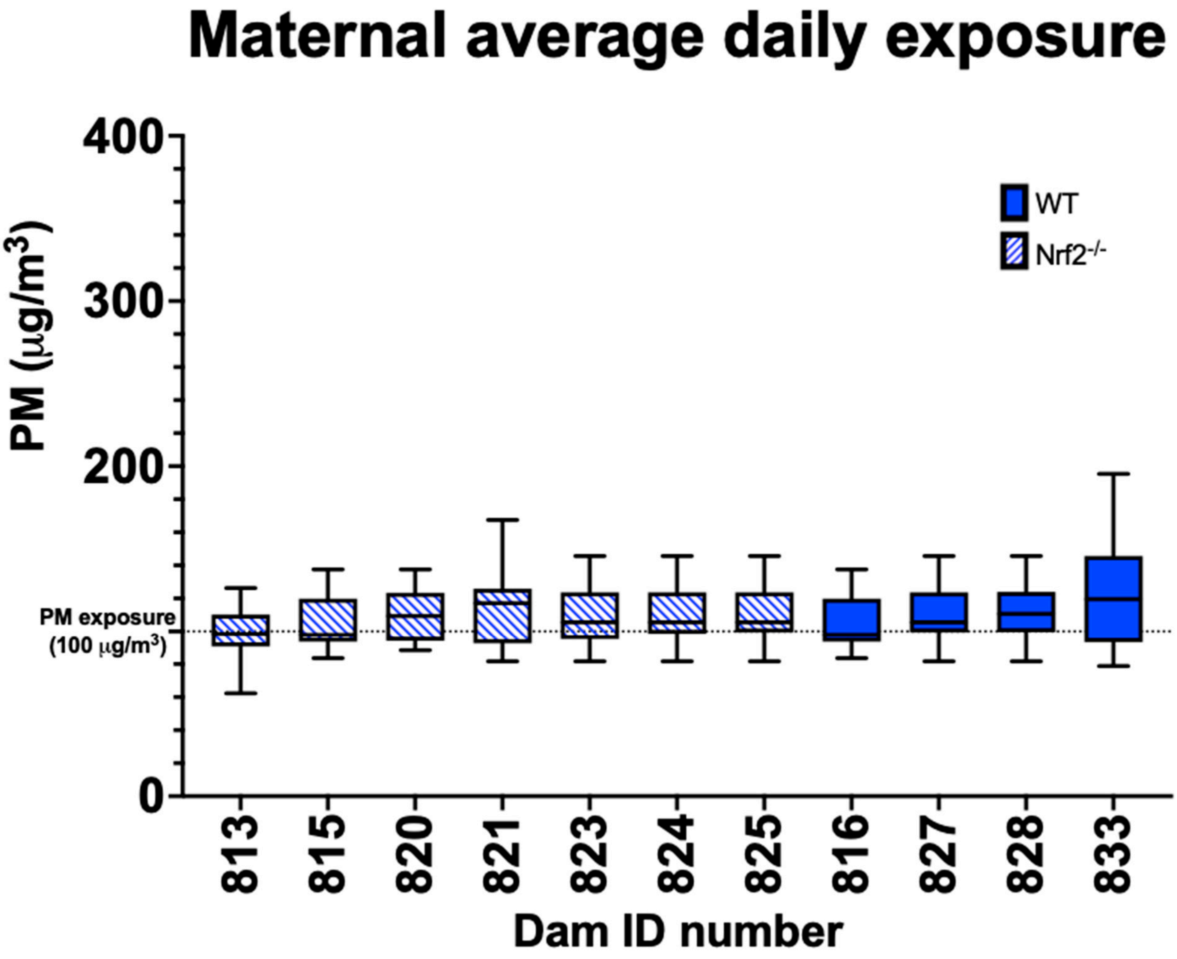

Supplemental Figure S1: Average maternal exposure to particulate matter (PM) in the low dose (LD) averaged 111.88±4.40 μg/m^3. Error bars represent SD.

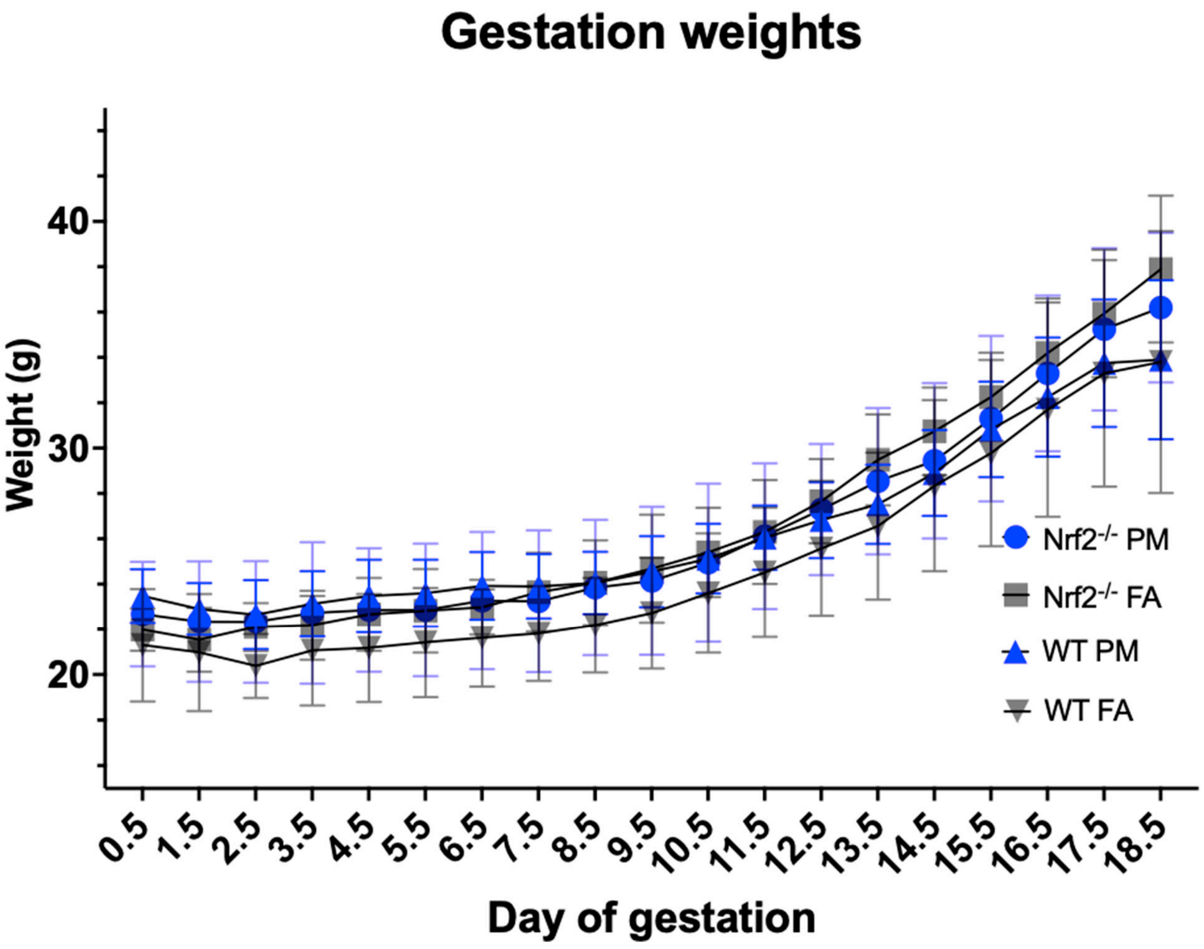

Supplemental Figure S2: Maternal weight gain during gestation did not significantly differ within or between exposure groups, with bars representing SD. Dam sample size includes WT FA (n=5), WT PM (n=4), *Nrf2*$^{-/-}$ FA (n=5), and *Nrf2*$^{-/-}$ PM (n=7).

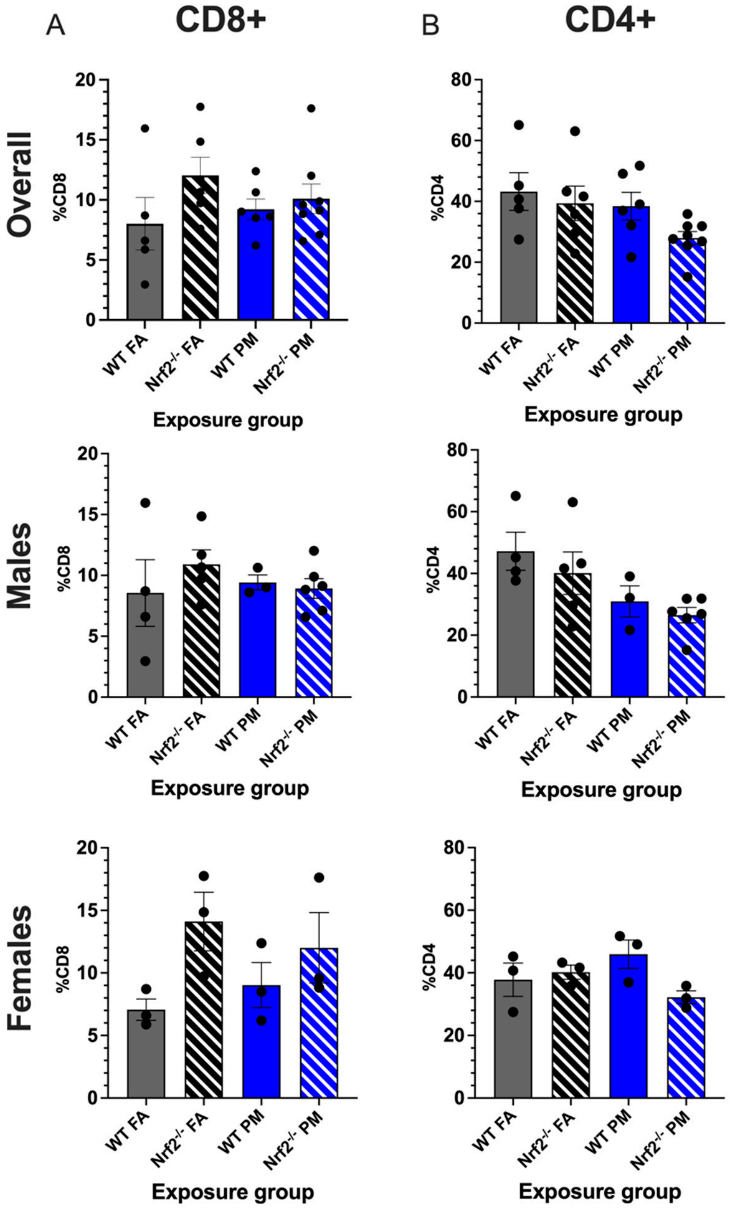

Supplemental Figure S3: CD8+ (A) and CD4+ (B) cells separated by sex. No significant differences are noted, though the overall level of CD4+ cells appears slightly lower in PM-exposed *Nrf2*$^{-/-}$ neonates (males and females) and WT neonates (males). Offspring sample sizes, listed as (n=Male, Female), from 4-7 litters, include WT FA (n= 3,3), WT PM (n=3,3), *Nrf2*$^{-/-}$ FA (n=5,3), and *Nrf2*$^{-/-}$ PM (n=6,3). Error bars represent SEM. Data analyzed using two-way ANOVA with Tukey's multiple comparison test.

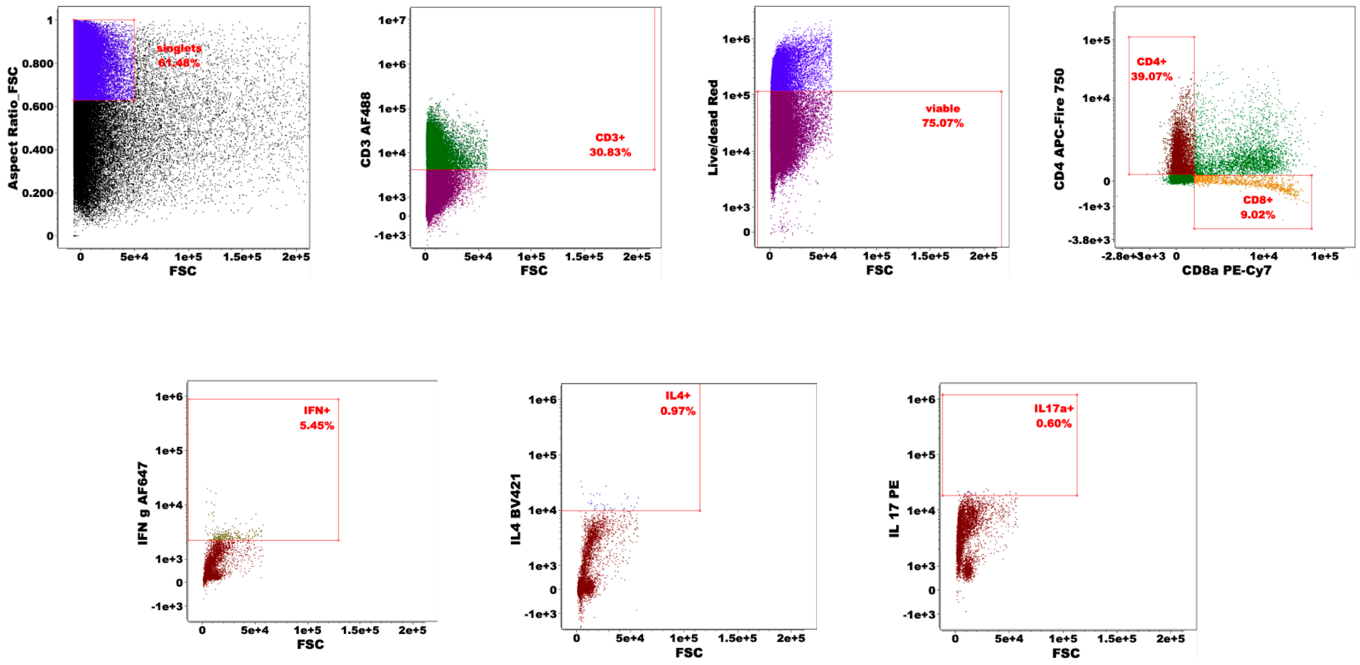

Supplemental Figure S4: CD4+ subsets gating strategy, FA sample. Gating from left to right, top to bottom: Singlets, Viable cells, CD3+ cells, CD4+ cells, IFN-γ, IL-4, and IL-17a.

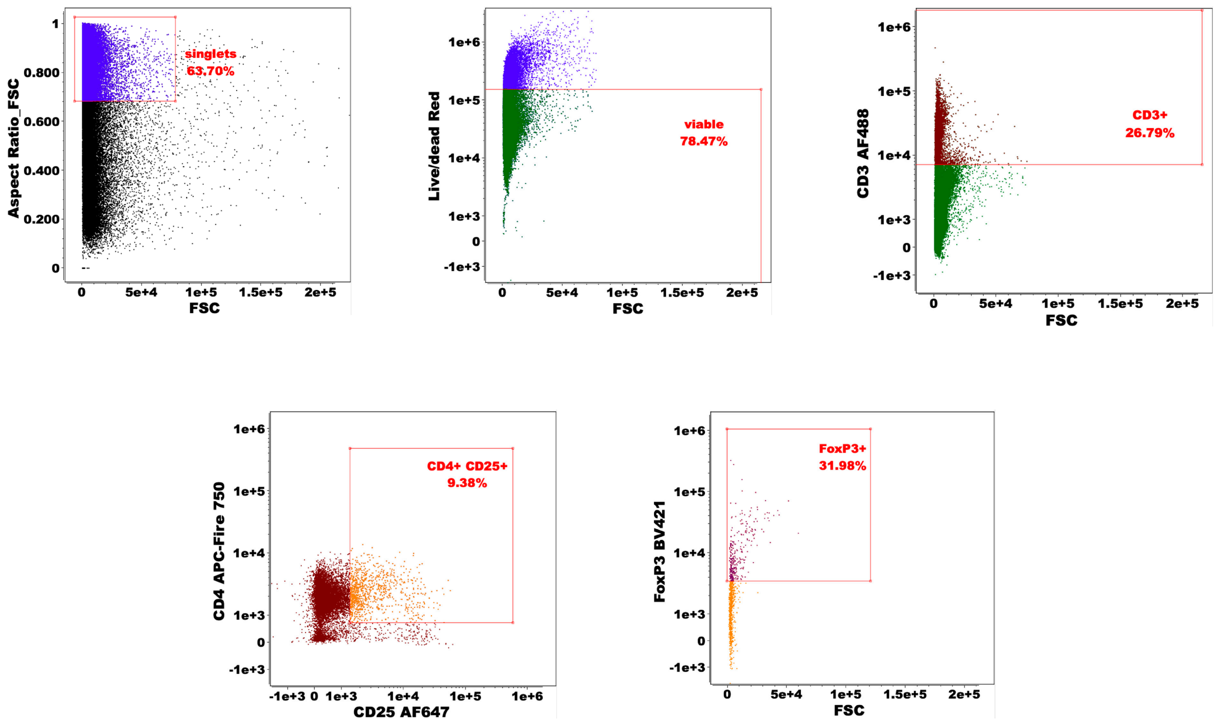

Supplemental Figure S5: T regulatory gating strategy, FA sample. Gating from left to right, top to bottom: Singlets, Viable cells, CD3+ cells, CD4+CD25+ cells, FoxP3+ cells.

Table S1: Oxidative stress biomarkers depicting cysteine (CyS), cystine (CySS), glutathione (GSH), and glutathione disulfide (GSSG) values in neonatal livers at PND5, represented as mean ± SEM.

	Overall				Male				Female			
	**WT FA**	**WT PM**	**Nrf2 FA**	**Nrf2 PM**	**WT FA**	**WT PM**	**Nrf2 FA**	**Nrf2 PM**	**WT FA**	**WT PM**	**Nrf2 FA**	**Nrf2 PM**
**GSH**	72.87±12.23	72.87±9.33	43.33±9.91	47.91±4.32	91.95±19.84	82.84±8.96	56.28±18.25	47.14±5.57	53.79±9.88	62.89±16.17	30.38±4.95	48.68±7.26
**GSSG**	33.08±2.95	29.41±1.89	24.65±3.18	25.50±0.84	37.53±4.70	31.70±1.82	26.46±6.44	24.75±1.68	28.64±2.14	27.11±3.19	22.84±1.58	26.25±0.27
**CyS**	1.12±0.25	1.82±0.63	0.96±0.40	0.70±0.08	1.42±0.40	2.01±0.99	1.43±0.78	0.64±0.12	0.82±0.29	1.64±0.90	0.50±0.07	0.76±0.12
**CySS**	2.48±0.35	2.51±0.19	2.41±0.23	2.76±0.26	2.98±0.58	2.83±0.32	2.50±0.43	2.13±0.14	1.97±0.27	2.19±0.11	2.32±0.23	3.39±0.31

Supplementary Methods

Genotypes of homozygous wild-type and *Nrf2*-deficient mice were confirmed by PCR amplification of genomic DNA extracted from tail snips. PCR amplification was carried out using established methods (Itoh et al. 1997) by using three different primers, 5′-TGGACGGGACTATTGAAGGCTG-3′ (sense for both genotypes), 5′-CGCCTTTTCAGTAGATGGAGG-3′ (antisense for wild type), and 5′-GCGGATTGACCGTAATGGGATAGG-3′ (antisense for LacZ). Conditions were as follows, step 1 95°C 180 sec, step 2 95°C 30 sec, step 3 70°C 30 sec, step 4 72°C 30 sec, repeat steps 2-4 for 35 total cycles, followed by step 5 72°C 120 sec. Wild-type and mutant PCR products detected at 200-300 bp and 400 bp, respectively.

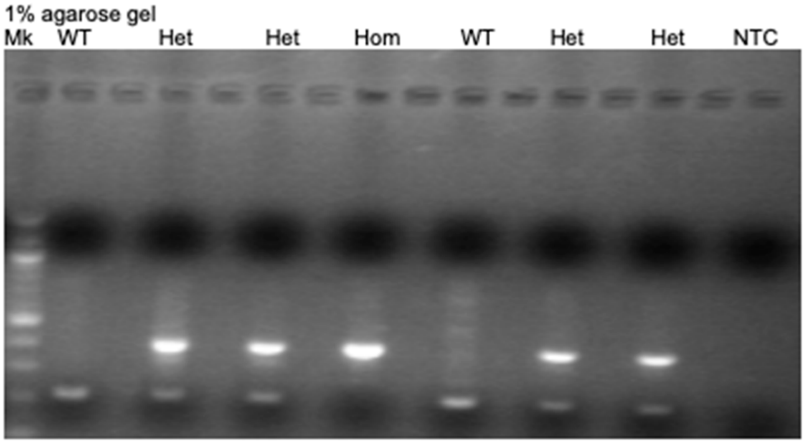

Reference:

Itoh, K., et al., *An Nrf2/Small Maf Heterodimer Mediates the Induction of Phase II Detoxifying Enzyme Genes through Antioxidant Response Elements.* Biochemical and Biophysical Research Communications, 1997. 236(2): p. 313-322.
